# Circulating tumor cells: advancing personalized therapy in small cell lung cancer patients

**DOI:** 10.1002/1878-0261.13696

**Published:** 2024-07-02

**Authors:** Prajwol Shrestha, Steven Kao, Veronica K. Cheung, Wendy A. Cooper, Nico van Zandwijk, John E. J. Rasko, Dannel Yeo

**Affiliations:** ^1^ Li Ka Shing Cell and Gene Therapy Program, Faculty of Medicine and Health University of Sydney Camperdown Australia; ^2^ Precision Oncology Program, Gene and Stem Cell Therapy Program, Centenary Institute University of Sydney Camperdown Australia; ^3^ Medical Oncology Calvary Mater Newcastle Waratah Australia; ^4^ Faculty of Medicine and Health University of Sydney Australia; ^5^ Medical Oncology Chris O'Brien Lifehouse Camperdown Australia; ^6^ Department of Tissue Pathology and Diagnostic Oncology, NSW Health Pathology Royal Prince Alfred Hospital Camperdown Australia; ^7^ School of Medicine University of Western Sydney Australia; ^8^ Cell and Molecular Therapies, Royal Prince Alfred Hospital Sydney Local Health District Camperdown Australia; ^9^ Concord Repatriation General Hospital, Sydney Local Health District Concord Australia

**Keywords:** DLL3, liquid biopsy, schlafen11, targeted therapies, tarlatamab, thoracic oncology

## Abstract

Small cell lung cancer (SCLC) is a highly aggressive cancer with a dismal 5‐year survival of < 7%, despite the addition of immunotherapy to first‐line chemotherapy. Specific tumor biomarkers, such as delta‐like ligand 3 (DLL3) and schlafen11 (SLFN11), may enable the selection of more efficacious, novel immunomodulating targeted treatments like bispecific T‐cell engaging monoclonal antibodies (tarlatamab) and chemotherapy with PARP inhibitors. However, obtaining a tissue biopsy sample can be challenging in SCLC. Circulating tumor cells (CTCs) have the potential to provide molecular insights into a patient's cancer through a “simple” blood test. CTCs have been studied for their prognostic ability in SCLC; however, their value in guiding treatment decisions is yet to be elucidated. This review explores novel and promising targeted therapies in SCLC, summarizes current knowledge of CTCs in SCLC, and discusses how CTCs can be utilized for precision medicine.

## Graphical abstract

Small cell lung cancer (SCLC) is an aggressive form of lung cancer that spreads rapidly to secondary sites such as the brain and liver. Cancer cells circulating in the blood, “circulating tumor cells” (CTCs), have demonstrated prognostic value in SCLC, and evaluating biomarkers on CTCs could guide treatment decisions such as for PARP inhibitors (schlafen11) and immunotherapies (PD‐L1 and DLL3).

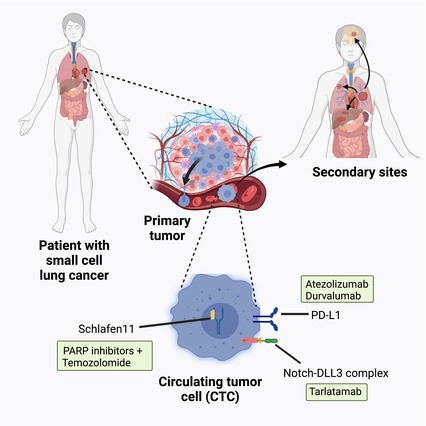

**Abbreviations**
ASCL1: achaete‐scute homolog 1
ATM: ataxia telangiectasia mutated
AURK: aurora kinase
Bcl2: B‐cell lymphoma 2
BiTE: bispecific T‐cell engager
bTMB: blood‐based tumor mutation burden
CHGA: chromogranin A
Chk1: checkpoint kinase 1
CK: cytokeratin
CPS: combined positive score
CSC: cancer stem cells
CTC: circulating tumor cell
ctDNA: circulating tumor DNA
DAPI: 4′,6‐diamidino‐2‐phenylindole
DLL3: delta‐like ligand 3
EBUS: endobronchial ultrasound
EMT: epithelial‐mesenchymal transition
EpCAM: epithelial cell adhesion molecule
EZH2: enhancer of zeste homolog 2
HR: hazard ratio
INSM1: insulinoma‐associated protein 1
LS‐SCLC: limited stage SCLC
MCL‐1: myeloid cell leukemia‐1
NE: neuroendocrine
NEUROD1: neurogenic differentiation factor 1
NGS: next generation sequencing
NSCLC: non‐small cell lung cancer
ORR: objective response rate
OS: overall survival
PARP: poly (ADP‐ribose) polymerase
PBMC: peripheral blood mononuclear cell
PCR: polymerase chain reaction
PD‐L1: programmed cell death protein ligand‐1
POU2F3: POU class 2 homeobox 3
pro‐GRP: pro‐gastrin‐releasing‐peptide
ROR1: receptor tyrosine kinase‐like orphan receptor 1
Rova‐T: rovalpituzumab tesirine
SCLC: small cell lung cancer
SLFN11: schlafen‐11
SYP: synaptophysin
TEP: tumor educated platelet
TMB: tumor mutational burden
TTF1: thyroid transcription factor‐1
Vim: vimentin
YAP1: Yes‐associated protein 1

## 1 Introduction

Lung cancer remains one of the most common cancers and is the leading cause of cancer‐related deaths globally [1]. Small cell lung cancer (SCLC) comprises of 15% of all lung cancers and is considered one of the most aggressive human cancer [2]. Globally, there are approximately 250 000 patients diagnosed with SCLC, and at least 200 000 patients will succumb to their disease each year [3, 4]. There are limited treatment options available for SCLC patients. For many years, combination chemotherapy comprising platinum‐based drugs and etoposide has been the standard of care. Recently, immunotherapies atezolizumab and durvalumab were approved in addition to the combinational chemotherapies; however, survival increments were modest (2–3 months) [5]. Classically, SCLC patients initially show a favorable response to first‐line therapy (58–68%); however, these responses are short‐lived [6]. Recent clinical trials have demonstrated promising outcomes for novel therapeutic strategies such as bispecific T‐cell engager antibodies (BiTE: tarlatamab) [7] and the synergistic combination of PARP inhibitors with temozolomide (TMZ) chemotherapy [8]. Additionally, subtyping based on transcriptomic gene signatures could select patients likely to benefit from immunotherapy [9].

Tissue biopsies are challenging to obtain in SCLC and are not routinely undertaken following diagnosis [10]. As such, liquid biopsies, specifically circulating tumor cells (CTCs) that represent *bona fide* tumor cells within the blood, may provide a convenient and suitable alternative. This review summarizes the existing evidence for CTCs in SCLC, including their prognostic value, and explores their potential role in clinical treatment selection and therapeutic response prediction. CTCs in SCLC may be used to evaluate the expression of tumor biomarkers such as delta‐like ligand 3 (DLL3) and schlafen11 (SLFN11) for precision medicine.

### 1.1 SCLC diagnosis

Small cell lung cancer has neuroendocrine (NE) characteristics, and pathological diagnosis is based on either (classic) histologic or cytologic appearance combined with the expression of specific immunohistochemical (IHC) markers. A panel of immunohistochemical markers is required for a definitive diagnosis of SCLC. Low‐molecular‐weight cytokeratins (CK; AE1/AE3 antibodies) or CK8 (cam5.2 antibody) are often observed in SCLC, typically in a dot‐like pattern. NE markers are also used, where CD56 is the most sensitive, followed by synaptophysin and chromogranin. However, all 3 NE markers can be negative, so insulinoma‐associated protein 1 (INSM1) may be used in addition [11, 12]. Thyroid transcription factor‐1 (TTF‐1) is often expressed (80–90%) regardless of pulmonary or extrapulmonary origin [13]. For tumors lacking NE markers and TTF1, it is critical to demonstrate negative expression of squamous cell markers such as p40 and CK5/6 to exclude basaloid squamous cell carcinoma. For CK‐negative tumors, exclusion of non‐epithelial small round cell malignancies such as lymphoma, melanoma, or sarcoma is required. Non‐specifically, the proliferative marker Ki67 is highly expressed (> 50%) in SCLC [14]. p16 is not often used due to non‐small cell lung cancer (NSCLC) also expressing p16 (40–50%).

Acquiring sufficient tissue for a pathological diagnosis can be challenging. The central tumor location and the presence of tumor necrosis can frequently complicate the process of obtaining a reliable diagnosis [10]. Additionally, frail patients may not be ideal candidates for endoscopic procedures like endobronchial ultrasound (EBUS) [15]. In cases where patients present with pleural effusion, thoracocentesis is a valuable diagnostic tool, and the diagnosis can be confirmed by examination of cells obtained from pleural fluid [16]. As SCLC is characterized by rapid progression and a rapid tumor doubling time as short as 38 days [17, 18], the initiation of systemic therapy is commenced as soon as possible after diagnosis. As such, a quick and definitive pathological diagnosis in SCLC is important and may be achieved through a liquid biopsy.

### 1.2 Liquid biopsy in SCLC

**Fig. 1:**
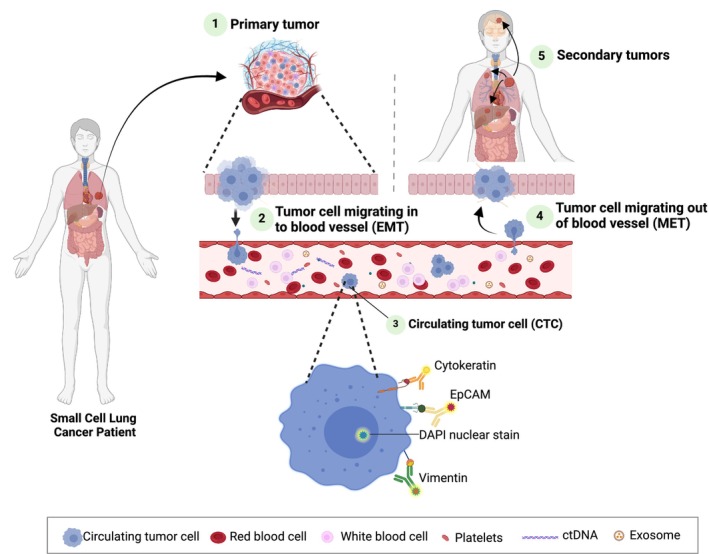
Metastatic seeding in small cell lung cancer (SCLC). Primary SCLC (1) where cancer cells extravasate and undergo epithelial‐mesenchymal transition (EMT) to enter the bloodstream (2). The cancer cell/s travel through the bloodstream, termed circulating tumor cells (CTCs) (3). Typical markers used for the detection of CTCs include cytokeratin, EpCAM, and vimentin with nuclear DAPI stain. CTCs undergo mesenchymal‐epithelial transition (MET) as they exit the bloodstream at the secondary site (4) where they grow to establish as a metastatic tumor (5).

In recent years, liquid biopsies have gained significant popularity as a research focus [19, 20]. Liquid biopsies may contain several types of tumor cells or their derivatives, such as circulating tumor DNA (ctDNA), CTCs, exosomes, microRNAs, tumor‐educated platelets (TEPs), and circulating tumor vascular endothelial cells (CTECs) [21]. Of these, ctDNA and CTCs have been the most extensively studied in SCLC patients. ctDNA are fragments of tumor DNA released into the peripheral blood, while CTCs are malignant cells that have detached from the tumor and circulate in the bloodstream (Fig. 1). The detection of ctDNA in peripheral blood uses highly sensitive techniques such as digital droplet polymerase chain reaction (ddPCR) and next‐generation sequencing (NGS). The predictive and prognostic value of ctDNA has been explored in SCLC, however, has limited clinical utility due to the lack of oncogenic driver mutations [22, 23]. This is in contrast to NSCLC, which has been found to benefit from ctDNA monitoring [24].

CTCs have been detected in 60–90% of SCLC patients, depending on the CTC platform used [25, 26, 27]. A recent meta‐analysis found that CTCs had prognostic potential in SCLC greater than NSCLC [28]. This makes CTCs an attractive tool for disease monitoring and molecular characterization in SCLC, serving as a potential surrogate for tissue biopsies [29].

#### 1.2.1 CTC detection

Accurate and reliable detection of CTCs remains a challenge, compounded by the heterogeneity of CTCs. Recent reviews have discussed different isolation methods for the detection of CTCs [30, 31, 32]. Briefly, methods to detect CTCs can be done either by size, density, or expression of surface markers. Microfluidic techniques like Parsortix and Clearcell use the differential size of PBMCs to separate CTCs from other blood cells. Alternatively, positive or negative selection methods identify CTCs by specific surface markers, such as CellSearch, which captures epithelial cell adhesion molecule (EpCAM)‐positive cells [33]. Recently, high‐resolution imaging‐based platforms such as high‐definition single cell assay (HDSCA) and AccuCyte‐CyteFinder have been developed without the requirement of positive or negative selection and rely on image analysis for the detection of positively stained CTCs using a panel of markers.

Circulating tumor cells can undergo epithelial‐mesenchymal transition (EMT) thereby reducing the expression of epithelial markers such as EpCAM and CK8/18/19 and increasing the expression of mesenchymal markers such as vimentin (Vim) and c‐MET [34, 35]. As such, epithelial markers used to detect CTCs may not detect those undergoing EMT. For example, 27% (6/22) of patients who had no detectable CTCs through CellSearch had detectable CTCs when analyzed with the addition of Ki67 and Vim markers [36]. Combinations of epithelial, endothelial, and mesenchymal expressions on SCLC CTCs, detected using HDSCA, illustrate the heterogeneous nature of CTCs [37]. Thus, better markers for SCLC CTCs can improve the accuracy of CTC detection and enumeration.

## 2 SCLC prognosis and CTCs

The prognostic value of CTCs has been demonstrated in several cancers, including SCLC [25, 26, 27, 38, 39, 40, 41, 42]. In SCLC, CTC counts at baseline and after the first cycle of chemotherapy were prognostic, regardless of the CTC detection method or CTC cut‐off value (Table 1). Naito et al. [25] found that a CTC baseline count of > 8 correlated with poorer survival. Hilterman et al. [26] demonstrated that the absolute CTC count after the first cycle of chemotherapy was the strongest predictor of survival, while Normanno et al. [27] found that a reduction of CTCs following chemotherapy had a stronger prognostic value than the absolute CTC count. In another study, CTC enumeration at cycle 2 was best associated with progression‐free survival (PFS) and overall survival (OS) [43]. A meta‐analysis of 16 studies found a high CTC number pre‐ and post‐therapy correlated with poor OS despite significant heterogeneity among the studies (*I* ^2^ = 81.8–87.1%) [44].

**Table 1:** Prognostic value of CTCs in SCLC. CK, cytokeratin; CTCs, circulating tumor cells; DAPI, 4′,6‐diamidino‐2‐phenylindole; EpCAM, epithelial cell adhesion molecule; ES, extensive stage; LS, limited stage; LT‐PCR, ligand‐targeted polymerase chain reaction; OS, overall survival; PFS, progression free survival; RT qPCR, real time quantitative PCR; SCLC, small cell lung cancer; Vim, vimentin.

Year [reference]	SCLC stage	*N*	CTC detection method (detection %)	Markers	Prognostic value	CTC cut off (per 7.5 mL blood)	Timepoint of CTC measurement (CTC cut‐off)
2009 [29]	LS/ES	50	CellSearch (86)	CK, CD56, DAPI, CD45, M65, M30	OS		Baseline and day 22 (1)
2012 [25]	LS/ES	51	CellSearch (69)	EpCAM, CD45	OS	≥ 6	Baseline and at relapse (6)
2012 [116]	LS/ES	97	CellSearch (85)	EpCAM, CK, CD45, DAPI	PFS OS	> 50	Baseline and after 1 cycle of chemotherapy (50)
2012 [26]	LS/ES	59	CellSearch (73)	EpCAM, CK, CD45, DAPI	PFS OS	2	Baseline (2)
2013 [117]	LS/ES	55	RT qPCR (78)	CK19	PFS OS	Not specified	Baseline, Post cycles 1 and 5 chemotherapy (not specified)
2014 [118]	LS/ES	30	TelomeScan: OBP‐401 assay (95)	GFP	OS	2	Baseline (2)
2014 [119]	LS	112	CellSearch (78)	EpCAM, CK8/18/19, DAPI	PFS OS	> 218 (Based on ROC curve)	Baseline (218)
2014 [27]	ES	60	CellSearch (90)	Not specified	OS		Baseline, Post one cycle of chemotherapy (Δ89%)
2017 [120]	LS/ES	80	LT‐PCR (84)	Folate receptor	PFS	–	Baseline
2017 [38]	ES	89	CellSearch (83.3)	CK, CXCR4, CD45, DAPI	PFS OS	≥ 6	Baseline and post cycle 1 chemotherapy (6)
2017 [43]	LS/ES	50	CellSearch (94)	EpCAM, γH2AX, M30	PFS OS	50	Baseline and post 1 cycle of chemotherapy (50)
2018 [121]	LS/ES	56	CellSearch (60)	CK, DAPI, Vim, Ki67, M30	PFS OS	≥ 5	Baseline, 1 cycle of chemotherapy and at disease progression (5)
2019 [39]	LS	75	CellSearch (60)	EpCAM, CK, DAPI	OS	15	Baseline (15)
2021 [21]	LS/ES	33	Negative selection, iFISH (97)	CEP8, CD44, CD45, Vim	PFS OS	≥ 12	Disease progression (12)
2022 [122]	LS/ES	21	CellSearch (86)	CK, EpCAM, DAPI	PFS	≥ 2, ≥ 150	Baseline (2)
2022 [53]	LS/ES	21	EpCAM positive selection (47.8), Parsortix (55)	CK, Vim	OS	≥ 50	Baseline (50)

## 3 CTC biomarkers for SCLC treatment

Biomarkers to identify patients for targeted therapies are expected to become important tools in medical oncology. However, in SCLC, there is a lack of actionable mutations or fusions [45]. As such, this section will discuss tissue biomarkers known in SCLC (Table 2): PD‐L1, tumor mutation burden (TMB), DLL3, SLFN11 and explore the evidence for their use on CTCs in SCLC and in other tumor types.

**Table 2:** Biomarkers in SCLC CTCs. ASCL1, achaite‐scute homolog 1; CHGA, chromogranin A; CK, cytokeratin; CTC, circulating tumor cell; DAPI, 4′,6‐diamidino‐2‐phenylindole; DLL3, delta‐like ligand 3; EpCAM, epithelial cell adhesion molecule; IF, immunofluorescence; NEUROD1, neurogenic differentiation factor 1; OS, overall survival, PD‐L1, programmed cell death protein ligand‐1; PFS, progression free survival; POU2F3, POU class 2 homeobox 3; SCLC, small cell lung cancer; SLFN11, schlafen‐11; SYP, synaptophysin; Vim, vimentin; YAP1, Yes‐associated protein 1.

Biomarker	Year [reference]	Assay	*N*	CTC platform (CTC markers)	CTC findings	
					Biomarker expression %	Prognostic/predictive relevance
PD‐L1	2016 [54]	IF (clone: SP142)	18	ISET system (not specified)	0 (42% on circulating immune cells)	–
	2017 [123]	IF (clone: EL1L3N)	6	Epic sciences (CK)	50	OS
	2021 [55]	Not specified	14	CellSave (EpCAM)	NA	Predicted response to platinum doublet reintroduction following myc inhibitor treatment
	2022 [53]	IF (clone: 28.8)	21	EpCAM positibe selection and Parsortix system (CK, CD16, CD66b, Vim)	7.7 (EpCAM positive selection); 9 (Parsortix)^a^	No OS difference
Bcl2	2018 [107]	IF (clone: 100/5)	66	CellSearch (CK, Vim, Bcl2, M30)	72.7	OS at baseline and post cycle 1
DLL3	2019 [82]	Taqman PCR	48	Parsortix System/qPCR (EpCAM, CK19, CHGA, SYP)	8.3	OS at baseline
	2019 [83]	IF (clone: A45‐B/B3)	108	CellSearch (CK, DLL3, Vim)	74.1	PFS at baseline and post cycle 1
SLFN11	2022 [91, 124]	IF (clone: D8W1B)	42	Epic sciences (CK)	45	Predicted clinical response
Molecular subtyping	2022 [100]	IF for ASCL1, NEUROD1, POU2F3, YAP‐1	28	AccuCyte‐CyteFinder (CK, EpCAM)	57.1 (ASCL1), 39.3 (NEUROD1), 42.9 (POU2F3), 32.1 (YAP‐1)	–

a % of total number of CTCs detected.

### 3.1 PD‐L1

Programmed cell protein‐1 (PD‐1) is expressed on T‐cells and, when engaged by its ligand, PD‐L1, inhibits the T‐cell activation pathway [46]. PD‐L1 staining on tissues has profoundly changed the treatment management for NSCLC, but in SCLC, it remains to be proven [47]. As such, PD‐L1 staining is not routinely undertaken. A number of clinical trials have examined PD‐L1 expression in SCLC; however, methods for measuring and quantifying positive PD‐L1 expression differ widely. The Keynote 028 study defined PD‐L1 positivity as > 1% of tumor cells, while the Keynote 158 study used a PD‐L1 combined positive score (CPS), which is the percentage of PD‐L1 positive cells (i.e., tumor cells, lymphocytes, and macrophages) of the total number of tumor cells. A pooled analysis of these studies assessing the efficacy of pembrolizumab beyond first‐line treatment of SCLC demonstrated a response rate of 29.7% (14/47) in PD‐L1‐positive patients compared to 3.5% (1/28) in PD‐L1‐negative patients [48]. PD‐L1 CPS expression was not helpful in predicting response to pembrolizumab with platinum doublet therapy using the IHC22C3 pharmDx assay (Keynote 604) [49]. Impower 133 demonstrated the benefit of adding atezolizumab to platinum doublet chemotherapy in extensive‐stage SCLC patients [50]. The study defined PD‐L1 expression as ≥ 1% and ≥ 5% present on either tumor cells or tumor‐infiltrating immune cells. Although a longer median OS in the PD‐L1 ≥ 5% group was observed with the addition of atezolizumab, significance was not reached due to the small numbers.

#### 3.1.1 PD‐L1 and CTCs

PD‐L1 expression in CTCs has been studied in various cancers, including SCLC [51, 52]. Acheampong et al. [53] reported PD‐L‐1 expression on CTCs isolated from 21 SCLC patients using the Parsortix microfluidic system or EpCAM‐positive selection. PD‐L1 was found to be positive in 7.7–9% of the total number of CTCs. Predictive analysis of PD‐L1 expression on CTCs was not feasible due to the limited number of patients. Ilie et al. [54] conducted a study in 18 SCLC patients and examined PD‐L1 on CTCs isolated by the ISET filtration‐based platform (Rarecells Diagnostics, Paris, France). PD‐L1 was not found on CTCs or in the tumor cells on the matched tissue but was observed in the tissue microenvironment and circulating immune cells. In a clinical trial investigating a Myc inhibitor, RRx‐001, PD‐L1 expression was examined on CTCs before and after therapeutic intervention [55]. A decrease in PD‐L1 expression in CTCs following RRx‐001 treatment was observed and was associated with a positive response when platinum doublet therapy was reintroduced, suggesting that PD‐L1 expression could potentially aid in selecting patients likely to respond.

### 3.2 Tumor mutational burden

Tumor mutational burden (TMB) has been examined in a number of studies in SCLC patients. In studies in patients with melanoma, NSCLC, and bladder cancer, TMB was found to be predictive of immunotherapy response, leading to FDA approval for pembrolizumab in TMB‐high cancers in 2020 [56, 57]. TMB is the total number of somatic mutations in DNA measured as mutations per megabase (Mut/Mb), where conventionally, ≥ 10 Mut/Mb is considered high [58]. It is measured in a coding area of the tumor genome and reflects all non‐synonymous coding mutations. TMB has been studied for predicting response to immunotherapy in SCLC; however, various TMB cut‐offs and methods have been used. In CheckMate 032, high TMB (≥ 248 mutations) was associated with a higher response rate in both treatment arms: nivolumab or combination nivolumab with ipilimumab; compared to tumors with the low TMB (< 144 mutations) (21.3% and 46% compared to 4.8% and 22.2%, respectively) [59, 60, 61]. The 1‐year OS was higher in the TMB high group (35% in the nivolumab arm and 62% in the combination arm) compared to TMB low group (22.1% and 23.4%, respectively). The difference was most prominent in the combination arm (median OS of 22 months in the TMB high group and 3.4 months in the TMB low group). Similar results were also found in Keynote 158, where pembrolizumab achieved an ORR of 29% with a response duration of 4.1–32.5 months in high TMB (defined by the conventional classification of ≥ 10Mut/Mb) [62]. In IMPower 133, TMB was measured using blood‐based TMB (bTMB), and 86% of SCLC patients had detectable bTMB, where < 10 bTMB and > 16 bTMB had an OS benefit with the addition of atezolizumab (hazard ratio (HR) = 0.69 and HR = 0.73, respectively) [50, 63].

#### 3.2.1 TMB and CTCs

Tumor mutational burden has not been studied in SCLC CTCs; however, studies have demonstrated the feasibility of testing TMB on CTCs in other cancer types. Rodriguez et al. [64] tested TMB in CTCs using low‐pass whole‐genome sequencing in 108 patients with prostate, breast, colorectal, bladder, and lung cancer. In a small study, Li et al. [65] compared whole‐exome sequencing data between NSCLC primary and progressive specimens of NSCLC, and an increase in TMB was observed after cancer progression.

### 3.3 Delta‐like ligand 3

**Fig. 2:**
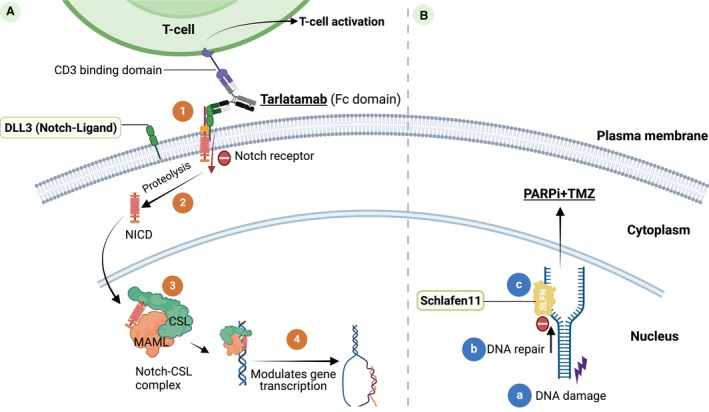
Emerging therapeutic targets and biomarkers in small cell lung cancer (SCLC). (A) Delta‐like ligand 3 (DLL3) belongs to the Notch signaling pathway family. DLL3 binds to the Notch receptor (1), which triggers protease‐mediated cleavage, resulting in the notch‐intracellular domain (NICD) being released into the cytoplasm (2). NICD migrates to the nucleus, where it binds to the transcription factor CBF1‐suppressor of hairless‐LAG1 (CSL) and recruits the transcription co‐factor mastermind‐like (MAML) (3) for gene transcription of Notch target genes (4). Targeted DLL3 therapy, tarlatamab (AMG757), is a bispecific T‐cell engager (BiTE) that binds to DLL3 on cancer cells and CD3 on T‐cells, resulting in T‐cell‐dependent killing. (B) Schlafen11 (SLFN11) is involved in the DNA damage repair pathway. Upon DNA damage or replication stress (a), DNA repair is initiated (b). SLFN11 binds to the replication fork (c) to prevent DNA damage repair and induce replication block and cell death. As such, SLFN11 can sensitize cells to DNA damage‐induced replication stress such as poly ADP ribose polymerase inhibitors (PARPi) combined with chemotherapy and temozlomide (TMZ). Therapeutic agents are underlined.

Delta‐like ligand 3 is a Notch ligand and has been extensively studied as a biomarker and a therapeutic target in SCLC [66, 67, 68, 69, 70, 71, 72, 73, 74, 75]. Notch signaling is “oncosuppressive” in SCLC (Fig. 2). The Notch pathway comprises four receptors (Notch 1–4) and five ligands (DLL1, 3–4, and Jagged 1–2). DLL3 is regulated by a transcription factor, achaete‐scute homolog 1 (ASH1), and acts as an oncogenic driver in SCLC by inhibiting Notch signaling [76]. DLL3 is highly expressed in SCLC and is not detectable in normal tissues [76]. ≥ 25% DLL3 tumor expression was found in 85% (895/1050) of patients using IHC staining, with 68% (719/1050) exhibiting high expression (≥ 75%) [77]. A meta‐analysis of five studies involving a total of 601 SCLC patients found that high DLL3 expression in tumor samples from extensive‐stage SCLC was significantly correlated with a poor prognosis, particularly in the Asian population [78].

Delta‐like ligand 3 targeting antibodies, rovalpituzumab tesirine (Rova‐T) and tarlatamab (AMG757), have been studied in relation to DLL3 expression [66, 67]. Rova‐T clinical trials did not demonstrate a survival benefit [68, 79]. DLL3 positivity (≥ 25%) or high DLL3 (≥ 75%) tumors did not correlate with response to Rova‐T, and cell lines derived from subjects that had progressed on Rova‐T did not show sensitivity to Rova‐T [80].

Tarlatamab is a bispecific T‐cell engager (BiTE) that binds to DLL3 on cancer cells and CD3 on T‐cells. This enhances the T‐cell‐dependent killing of cancer cells. The phase I study found manageable side effects and clear signs of activity in SCLC patients [73]. In a subsequent study involving 107 refractory or relapsed SCLC patients, an ORR of 23.4% was observed, including two complete responses and 23 partial responses [7]. The median duration of response at the time of reporting was 12.3 months and the median PFS was 3.7 months. Preliminary analysis suggests selecting patients with increased DLL3 expression provides enhanced clinical benefit. The phase II DeLLphi‐301 study (NCT05060016) involving 220 previously treated SCLC patients found an ORR of 32–40% and an estimated 9‐month overall survival of 66% [81]. Low DLL3 expression has been proposed as a resistance mechanism to DLL3‐targeting BiTEs [70]. Tarlatamab in combination with other agents such as an anti‐PD1 agent, AMG404 (NCT04885998), and chemotherapy and anti‐PD‐L1 combination (NCT05361395) are currently underway. Several other DLL3‐targeting agents, such as BI764532 [75], AMG119 [69], and HPN328 [72], are also under clinical investigation.

#### 3.3.1 DLL3 and CTCs

Delta‐like ligand 3 expression has been evaluated in SCLC CTCs. Obermayr et al. [82] reported the detection of DLL3 transcripts by the TaqMan assay in CTCs isolated by the Parsortix microfluidic platform as a marker of poor survival. Similarly, Messaritakis et al. [83] found DLL3 expression, by antibody staining, in CTCs at baseline was significantly associated with decreased PFS (HR = 10.8) and OS (HR = 28.2). 74.1% (80/108) of patients had DLL3‐positive CTCs and were not detected in any healthy controls. Of the 14 patients with high DLL3 tissue expression (> 50% tumor cells), 85.7% (12/14) had detectable DLL3‐positive CTCs. Interestingly, 83.3% (5/6) of patients with low DLL3 tissue expression had detectable DLL3‐positive CTCs, with the authors proposing the potential of systemic migration of DLL3‐positive tumor cells. The number of DLL3‐positive CTCs and the detection rate also significantly increased with disease progression. In a small study, 83.3% (5/6) of patients with CK‐negative CTCs were noted to have detectable DLL3‐positive and Vim‐positive CTCs [80]. DLL3 expression on CTCs in patients undergoing DLL3‐targeted treatments has so far not been examined as a predictive biomarker.

### 3.4 Schlafen11

SLFN11 is a DNA/RNA helicase required for chromatin opening that induces irreversible DNA replication block by changing chromatin structure at replication sites (Fig. 2) [84]. Fifty to sixty per cent of SCLC patients are SLFN11‐positive [8, 85, 86, 87]. SLFN11 expression was detected by IHC using commercially available antibody SLFN11 clones such as SA, D‐2SC and E‐4SC [88, 89, 90]. SLFN11 is also expressed in inflammatory cells, which can influence tissue‐based bulk RNA sequencing. SLFN11 has been found to be downregulated after treatment in SCLC patients [91, 92].

As SLFN11 is recruited to stalled replication forks, it can sensitize cells to DNA damage‐induced replication stress. SLFN11 has been associated with the sensitivity to topotecan in SCLC cell lines, suggesting its use as an emerging predictive biomarker [93]. High SLFN11 expression has been linked to sensitivity to poly ADP‐ribose polymerase (PARP) inhibitors [86, 90]. Temozolomide (TMZ) chemotherapy was found to synergize with PARP inhibitors in SCLC cell lines and mouse models [90]. This was demonstrated in a phase II study where the response rate was significantly higher when the PARP inhibitor, veliparib, was combined with TMZ compared to TMZ alone in recurrent SCLC (39% vs 14%) [8]. When archival tissue was available, SLFN11‐positive patients who received combination therapy had significantly higher PFS (5.7 vs 3.6 months) and OS (12.2 vs 7.5 months). SFLN11 expression has been observed to change following treatment, potentially with the emergence of resistance [86, 94]. Downregulation of SLFN11 has been demonstrated to be mediated by EZH2 during treatment resistance. Using xenograft SCLC models, the addition of EZH2 inhibitors was found to overcome chemotherapy resistance [92]. SLFN11 expression has also been shown to correlate with response to Topoisomerase 1 and 2 inhibitors such as etoposide combined with alkylating agents like cisplatin, and low SLFN11 expression has been linked to therapy resistance in other cancers [85, 95, 96].

#### 3.4.1 SLFN11 and CTCs

SLFN11 has been evaluated in CTCs in high‐grade NE cancers, including SCLC [91]. 83% (53/64) of patients had detectable CTCs, using a high‐resolution CTC imaging platform (Epic Sciences, San Diego, CA, USA). Of these patients, 55% (29/53) had SLFN11‐positive CTCs, and this detection rate was comparable to the tissue. In three of these patients, longitudinal samples were available, and overall CTC number and SLFN11‐positive CTCs were correlated with clinical response. This suggests the potential of SLFN‐11 as a prognostic and predictive marker in SCLC patients, especially due to its association with treatment resistance; however, further studies are required.

### 3.5 Transcriptomic and proteomics‐based SCLC classification

Small cell lung cancer is known to have significant heterogeneity and variation in treatment response. Recent molecular characterization has identified 4 distinct SCLC subtypes based on their differential expression of transcription factors: SCLC‐A, SCLC‐N, SCLC‐P, and SCLC‐I, which demonstrated prognostication and could assist in treatment selection [9, 97, 98]. Two NE subtypes, expressing high expression of Chromogranin A (CHGA) and Synaptophysin (SYP) were identified: SCLC‐A (expressing ASCL1) and SCLC‐N (expressing NEUROD1). These were the most common subtypes, comprising 51% and 23%, respectively. The SCLC‐P subtype (expressing POU2F3) was the least common (7%) and had the worst prognosis [9]. The fourth subtype, SCLC‐I, did not express any of the key transcription factors and was associated with an inflammatory gene signature with high expression of immune checkpoint molecules, such as CD274, PD‐L1, PDCD1, CTLA4, CD80, CD86, CD38, ID O1, TIGIT, C10orf54 (Vista), ICOS, and LAG3, as well as T‐cell‐attracting chemokines such as CCL5 and CXCL10. Furthermore, the SCLC‐I subtype expressed low levels of epithelial markers such as E‐cadherin and high levels of mesenchymal markers such as Vim and Axl. Interestingly, the previously reported subtype, SCLC‐Y, associated with Yes‐associated Protein 1 (YAP1) was not found to be a distinct subtype in this study but was found to be expressed in both SCLC‐P and SCLC‐I subtypes [99].

Importantly, there are therapeutic implications for these molecular subtypes. The Impower 133 study demonstrated significant OS benefit in the SCLC‐I subtype compared to other SCLC subtypes for the atezolizumab combined with chemotherapy treatment group (HR = 0.566) [50]. While the SCLC‐A and SCLC‐N subtypes showed a trend toward improvement in median OS with the addition of atezolizumab, there was no difference in the SCLC‐P subtype.

#### 3.5.1 Molecular subtypes and CTCs

Molecular subtyping CTCs into the four SCLC subtypes is feasible [100]. Kopparapu et al. used a panel of markers: ASCL1, POU2F3, NEUROD1, and YAP‐1 to characterize the CTCs detected using the high‐resolution imaging platform, AccuCyte‐CyteFinder (RareCyte, Seattle, WA, USA). SCLC‐A was the most common subtype on CTCs (80%), followed by SCLC‐P (67%), SCLC‐N (55%), and SCLC‐Y (50%). Interestingly, the study found CTCs from the same patient expressed multiple subtype markers, suggesting molecular intra‐patient heterogeneity and/or subtype switching.

### 3.6 Future perspectives

There are a number of promising biomarkers for commonly used therapies, such as TMZ, PARP inhibitors, and B‐cell lymphoma 2 (Bcl2) inhibitors: venetoclax and navitoclax. In addition, there are several encouraging therapies for SCLC, such as ROR1 inhibitors, aurora kinase inhibitors (AURKi), heat shock protein 90 (HSP90) inhibitors, and CDK9 inhibitors (dinaciclib). This section will explore these promising biomarkers and therapies and discuss how CTCs could be utilized for their translation.

#### 3.6.1 TMZ and PARP inhibitors

Patients with an inflammatory gene signature, SCLC‐I, benefited from the combination of a chemotherapy, TMZ, and a PARP inhibitor, talazoparib [101]. Absence of SCLC subtype markers (ASCL1, NEUROD1, POU2F3 or NE genes, and INSM1) and enrichment of non‐NE genes have been associated with increased EMT signatures in relapsed SCLC after first‐line platinum therapy [102], where both EMT and ataxia telangiectasia mutated (ATM) signatures could potentially help predict therapeutic response [86].

ATM and E‐cadherin have been investigated in SCLC xenograft models to study the activity of talazoparib. Low expression of ATM and checkpoint kinase 1 (CHK1) as well as high expression of SLFN11 were associated with a response to talazoparib, with high CHK1 expression associated with treatment resistance [86]. In castration‐resistant prostate cancer, ATM altered tumors were associated with SLFN11‐positive CTCs [103]. So far, there are no studies examining ATM expression in SCLC CTCs. If combined with SLFN11 expression, CTC examination could be of interest to determine sensitivity to PARP inhibitors.

#### 3.6.2 Bcl2, HDAC, and ROR1 inhibitors

In the SCLC‐A subtype, ASCL1 targets the antiapoptotic regulator, Bcl2, inducing cell death through the mitochondrial apoptotic pathway. In SCLC xenografts and cell lines, high levels of Bcl2 were associated with the treatment response to the Bcl2 inhibitor, navitoclax (ABT‐263) [104, 105]. Navitoclax did not produce a therapeutic benefit in SCLC patients, however, Bcl2 expression was not quantified in patients [106]. Bcl2‐positive CTCs were found in 72.6% of SCLC patients and were significantly associated with worse PFS (HR = 4.5) and OS (HR = 4.3) [107]. Bcl2 expression has been linked to plasma pro‐gastrin‐releasing peptide (pro‐GRP), and this has been correlated with sensitivity to navitoclax [106].

Bcl2 inhibitors have been used in combination with other therapies. Bcl2 inhibitors combined with a HDAC inhibitor, vorinostat, demonstrated improved activity including in Bcl2 resistant cell lines [108]. Another anti‐apoptotic protein from the Bcl2 family, myeloid cell leukemia‐1 (MCL‐1), has been shown to be highly expressed in SCLC patients [109]. The study found high MCL‐1 expression was associated with low Bcl‐xL expression, and the combination of a MCL‐1 inhibitor with navitoclax resulted in increased anti‐tumor killing. Venetoclax combined with either doxorubicin or a CDK9 inhibitor, dinaciclib, demonstrated significant anti‐tumor activity in SCLC xenograft models [110].

Bcl2 is co‐expressed with receptor tyrosine kinase‐like orphan receptor 1 (ROR1) in SCLC. ROR1, an oncofetal protein, is highly expressed in SCLC (93% by IHC, 79% by RT‐qPCR) [111]. A small‐molecule ROR1 inhibitor was found to have good anti‐tumor activity in SCLC‐derived cell lines and, when combined with venetoclax, demonstrated synergistic inhibition [111]. Examining MCL‐1 or ROR1 on SCLC CTCs could act as a predictive biomarker for MCL‐1 or ROR1 inhibitors combined with Bcl2 inhibitors.

#### 3.6.3 Proteomic SCLC subgroups and AURK, BCL2, and HSP90 inhibitors

Protein biomarkers show direct pathway activation and (protein) target expression. An analysis of plasma‐based proteins in SCLC patients found enrichment for Myc and YAP1 [112].

Myc is a known oncoprotein overexpressed in SCLC [96]. High levels of Myc have been found in the SCLC‐N subtype, and Myc expression resulted in sensitivity to aurora kinase (AURK) inhibitors in SCLC cell lines [9, 113, 114]. Myc and TTF‐1 are known targets of the transcription factors ASCL1 and NEUROD1 [115]. Two major SCLC proteomic subgroups were proposed based on model‐based clustering: high TTF‐1/low Myc and low TTF1/high Myc. The levels of TTF‐1 or Myc could predict treatment responses to AURK, Bcl2, or heat shock protein 90 (Hsp90) inhibitors [113].

## 4 Conclusions

Considering CTCs as an easily accessible tumor surrogate, they are well suited to be used as a companion diagnostic for biomarker‐guided therapy. However, large prospective studies are required to confirm their benefit before implementing for routine clinical care. In SCLC, a critical advantage of liquid biopsies is the ability to undertake serial sampling. This is vital, as rapid tumor progression on therapy is characteristic of SCLC. Several studies have demonstrated the prognostic value of CTCs and shown the feasibility of evaluating the expression of therapeutic targets on CTCs such as SLFN11 and DLL3. However, these biomarker studies were relatively small and were retrospective. As such, the potential for evaluating SCLC markers on CTCs as a means for treatment selection and predicting response requires further validation. A number of biomarker‐driven clinical trials are currently on‐going in SCLC: NCT03699956, NCT04334941, PRIO‐NCT04728230, and NCT04334941.

Before becoming clinically validated, CTC isolation and biomarker evaluation should be standardized and validated in prospective clinical trials. Several studies have explored PD‐L1 expression in tissue and CTCs, but variation in the staining techniques and scoring is confounding the interpretation of results. Therefore, it is crucial to assess the practical implications of CTC‐biomarker testing (including cost) and strive toward a general consensus on how to most effectively use CTC detection platforms as clinical tools. The application of CTCs for the assessment of biomarkers, in combination with transcriptomic‐based subtyping and proteomic characterization of SCLC, presents a unique opportunity to personalize treatment approaches to improve SCLC patient outcomes.

## Conflict of interest

JEJR reports advisory roles in Gene Technology Technical Advisory Committee, Office of the Gene Technology Regulator, Australian Government; and Human Research Ethics Committee, Genea. JEJR also reports honoraria speaker fees or advisory roles for SPARK Therapeutics, Cynata, and Pfizer Inc.; Woke Pharmaceutical (shares); Kennerton Capital (non‐executive director); AAVec Bio (co‐founder); consultant role for Rarecyte (stocks in lieu). SK served on advisory boards for AstraZeneca, Pfizer, MSD, BMS, Roche, Amgen, BeiGene, and Daiichi Sankyu. SK received honorarium (partly to institution) from MSD, BMS, Roche, AstraZeneca, Pfizer, Takeda, and BeiGene. SK received research grant (to institution) from AstraZeneca. All the other authors declare that the research was conducted in the absence of any commercial or financial relationships that could be construed as a potential conflict of interest.

## Author contributions

All authors (PS, SK, VKC, WAC, NZ, JEJR, and DY) were involved in the conceptualization, writing of the original draft, and reviewing and editing the manuscript. PS and DY created the figures. All authors have read and agreed to the published version of the manuscript.

## Data Availability

Data sharing is not applicable to this article. No new data were created or analyzed in this study.
